# Report of the WHO technical consultation on the evaluation of respiratory syncytial virus prevention cost effectiveness in low- and middle-income countries, April 7–8, 2022

**DOI:** 10.1016/j.vaccine.2023.09.040

**Published:** 2023-11-22

**Authors:** Meagan C. Fitzpatrick, Rachel S. Laufer, Ranju Baral, Amanda J. Driscoll, Daniel R. Feikin, Jessica A. Fleming, Mark Jit, Sonnie Kim, Mihaly Koltai, You Li, Xiao Li, Harish Nair, Kathleen M. Neuzil, Clint Pecenka, Erin Sparrow, Padmini Srikantiah, Justin R. Ortiz

**Affiliations:** a Center for Vaccine Development & Global Health, 685 W. Baltimore St., University of Maryland School of Medicine, Baltimore, MD 21201, USA; b Center for Vaccine Innovation and Access, PATH, 2201 Westlake Avenue, Suite 200, Seattle, WA 98121, USA; c World Health Organization, 20 Avenue Appia, Geneva 1211, Switzerland; d Centre for Mathematical Modelling of Infectious Diseases, London School of Hygiene & Tropical Medicine, London, UK; e Department of Infectious Disease Epidemiology, Faculty of Epidemiology and Population Health, London School of Hygiene & Tropical Medicine, London, UK; f National Institute of Allergy and Infectious Diseases, National Institutes of Health, Bethesda, MD, USA; g School of Public Health, Nanjing Medical University, Nanjing, China; h Centre for Global Health, Usher Institute, University of Edinburgh, Edinburgh, UK; i Centre for Health Economics Research & Modelling Infectious Diseases (CHERMID), Vaccine & Infectious Disease Institute, University of Antwerp, Belgium; j Centre for Global Health, Usher Institute, Edinburgh Medical School, University of Edinburgh, UK; k MRC/Wits Rural Public Health and Health Transitions Research Unit (Agincourt), School of Public Health, Faculty of Health Sciences, University of the Witwatersrand, Johannesburg, South Africa; l Bill & Melinda Gates Foundation, Seattle, USA

**Keywords:** Cost effectiveness, Global health, Monoclonal antibody, Respiratory syncytial virus, Vaccine

## Abstract

Policymakers often rely on impact and cost-effectiveness evaluations to inform decisions about the introduction of health interventions in low- and middle-income countries (LMICs); however, cost-effectiveness results for the same health intervention can differ by the choice of parameter inputs, modelling assumptions, and geography. Anticipating the near-term availability of new respiratory syncytial virus (RSV) prevention products, WHO convened a two-day virtual consultation in April 2022 with stakeholder groups and global experts in health economics, epidemiology, and vaccine implementation. The objective was to review methods, parameterization, and results of existing cost-effectiveness analyses for RSV prevention in LMICs; identify the most influential inputs and data limitations; and recommend and prioritize future data gathering and research to improve RSV prevention impact estimates in LMICs. Epidemiological parameters identified as both influential and uncertain were those associated with RSV hospitalization and death, specifically setting-specific hospitalization rates and RSV-attributable death rates. Influential economic parameters included product price, delivery costs, willingness-to-pay for health on the part of potential donors, and the cost of RSV-associated hospitalization. Some of the influential parameters identified at this meeting should be more precisely measured by further research. Other influential economic parameters that are highly uncertain may not be resolved, and it is appropriate to use sensitivity analyses to explore these within cost-effectiveness evaluations. This report highlights the presentations and major discussions of the meeting.

## Highlights

• Respiratory syncytial virus (RSV) is an important pathogen globally.
• The burden of RSV illness is highest in low/middle-income countries (LMICs).
• In April 2022, WHO convened a meeting to discuss the economics of RSV prevention.
• We reviewed cost-effectiveness analyses of RSV prevention in LMICs.
• We provided recommendations for future data gathering to address data limitations.

## 1 Background

Respiratory syncytial virus (RSV) is a leading cause of hospitalization in infants and young children due to lower respiratory tract illness (LRTI), including pneumonia and bronchiolitis; however, licensed preventive interventions and leading pipeline candidates are not anticipated to be affordable for low-income countries without subsidies; [1], [2], [3]. In 2016, recognizing the growing pipeline of RSV prevention products, the World Health Organization (WHO) Strategic Advisory Group of Experts on Immunization (SAGE) requested that preparations be made to support global policymaking for RSV preventive interventions [4]. To inform decisions about the introduction of RSV immunization products, policymakers in low- and middle-income countries (LMICs) will need to consider their impact and cost-effectiveness.

WHO convened an online meeting in April 2022 to review cost-effectiveness analyses for RSV prevention. The objectives of the meeting were the following: 1) to review objectives, methods, inputs, and results of cost-effectiveness analyses of RSV prevention for young children in LMICs; 2) to identify the most influential parameter inputs and data limitations for the cost-effectiveness analyses; and 3) to recommend and prioritize future data gathering and research to improve RSV prevention impact estimates in LMICs. Attendees included stakeholder groups and global experts in health economics, epidemiology, and vaccine implementation. The agenda and list of participants are in the Online Supplement.

## 2 RSV disease overview

RSV is a common respiratory virus that circulates in seasonal epidemics [5]. Its symptoms are usually mild and self-limited [6]. However, RSV can also cause severe disease. It is the most common cause of LRTI in young children globally [7], it can exacerbate chronic medical conditions, and it can cause acute respiratory illness in older adults [8]. RSV transmission can occur by contact or inhalation of airborne virus. Most individuals have evidence of RSV infection by two years of age [6], however subsequent reinfection is possible [9]. Among children, the greatest risk of severe RSV disease occurs in infants <6 months of age and in children with congenital heart disease or lung disease [6].

As of September 2023, there are no licensed vaccines administered to children for RSV prevention [2]. Clinical trials assessing pediatric RSV vaccine candidates in the 1960s were halted due to evidence of vaccine-associated enhanced disease [10], [11]. This safety signal slowed RSV vaccine development for decades. Since 1998, palivizumab, a humanized monoclonal antibody (mAb) directed against the F protein of RSV, has been licensed for use in young children at high risk for RSV disease [12]. The immunoprophylaxis is administered by monthly intramuscular injection throughout the RSV season [12]. Palivizumab is too expensive for use in most LMICs. Acknowledging that RSV preventive interventions are an unmet global health need, biomedical research funders including the US National Institutes of Health and the Bill & Melinda Gates Foundation have made substantial investments in understanding and preventing RSV disease. There is now a robust research and development pipeline for RSV prevention products, including monoclonal antibody (mAb) immunoprophylaxis and vaccines in late-stage development. By September 2023, extended half-life mAb have achieved licensure in some high income countries [13], [14] [15], maternal RSV vaccines have been licensed in Europe and in the United States [16] [17], and RSV vaccines for older adults have achieved licensure in Europe and the United States [18] [19].

While RSV prevention products are likely to become available first in high-income countries, efforts are underway to accelerate their availability and programmatic suitability in LMICs [1], [2]. A major requirement to justify funding is product cost-effectiveness, defined as the expenditure necessary to achieve a unit of health or other benefit. Cost-effectiveness is often an explicit part of decisions by regulatory bodies, countries, and donors about whether to adopt a health intervention. For instance, SAGE includes cost-effectiveness as one of the criteria considered when deciding whether to recommend vaccines for use [20], recommendations which are regarded as authoritative by many countries. Gavi, the Vaccine Alliance, is a major donor supporting immunization efforts for LMICs and lists “Value for Health” among its own criteria when considering which products to financially support [21]. For Gavi-eligible countries, adoption of a vaccination program is often conditional on both a SAGE recommendation and Gavi support, with additional country-specific considerations regarding the cost-effectiveness of the new intervention relative to current and potential uses of the health budget [22].

## 3 Disease burden

In 2022, researchers published an updated systematic analysis of global disease burden estimates for acute RSV LRTI in young children [7], [23]. The update included disease burden estimates within narrow age bands to facilitate impact modelling of potential RSV preventive interventions expected to have limited durations of protection [1], [2], [3]. Global and regional estimates of RSV community morbidity and hospitalization were presented, as well as RSV in-hospital and overall mortality burden from published and unpublished data, using a generalized linear mixed-effect modelling framework.

The research highlighted the substantial RSV morbidity and mortality burden in infants < 6 months, accounting for 20% and 45% of RSV LRTI episodes and deaths in children < 5 years, respectively. In LMICs, the RSV LRTI incidence rate was three times as high as that in high-income countries in the community whereas the RSV LRTI hospitalization rate was lower than that in high-income countries among infants < 6 months, highlighting the limited access to healthcare in LMICs. This was further emphasized by estimates for the RSV community mortality burden, which showed that 82% of RSV-attributable deaths occurred out of hospital and the infant case fatality ratio (CFR) of RSV LRTI in the community could be as high as 6.6% in low-income countries. These findings suggest that RSV immunization programs targeting protection during the first six months of life could have a substantial effect on reducing severe RSV disease burden. In LMICs, RSV immunization programs are likely to be even more impactful given that a considerable proportion of RSV morbidity and mortality was due to limited access to health-care services, and therefore these deaths could potentially only be averted through immunization programs. However, substantial year to year variability as well as intra- and inter-region variability in RSV morbidity and mortality (in a given year) were noted. In an attempt to attribute cause of death to the RSV related mortality estimate, two sets of estimates were presented – one where RSV was identified in the upper airway samples of a deceased child (RSV associated mortality); and the other where RSV was deemed to be in the causal chain based on the opinion of an expert adjudication panel, such as in CHAMPS (RSV attributable mortality) [24]. Although the most recent RSV mortality estimates incorporate more data on mortality than previous estimates, more data are needed to better characterize RSV mortality, particularly in community settings.

During the WHO meeting, RSV LRTI morbidity and mortality incidence estimates from the systematic review were compared with estimates determined by other high-quality studies, including mAb and vaccine trials and large, multi-country observational studies (Table 1). Estimates of several RSV LRTI epidemiologic parameters from the systematic analysis were similar to placebo arms in RSV intervention field trials, including RSV LRTI incidence in the first 3 and 6 months of life, and severe and hospitalized RSV LRTI incidence in the first 3 months of life [25], [26]. Severe RSV LRTI incidence estimates from the first two months of life were comparable to the findings of the Aetiology of Neonatal Infections in South Asia (ANISA) observational cohort study [27]. In-hospital CFR estimates for RSV LRTI among children < 5 years of age were similar to the Pneumonia Etiology Research for Child Health (PERCH) case control study [28], [29]. Incidence of RSV LRTI illnesses and hospitalizations during the first six months of life were appreciably lower in the systematic review (when restricted to LMICs) than in the placebo arm of an RSV mAb trial among US indigenous populations [30], possibly reflecting lower testing rates and worse access to care in LMIC compared to the US, even in underserved populations. The systematic review estimated much higher RSV LRTI morbidity and mortality during early childhood than the Institute for Health Metrics and Evaluation (IHME) Global Burden of Disease estimates in 2016 (33 million episodes and 101,000 deaths in review compared to 11 million cases and 41,000 deaths by IHME) [31].

**Table 1:** Comparison of RSV morbidity and mortality burden estimates between the 2022 RSV LRTI systematic review and other important studies^a.^

Parameter^b^	Study	Population	Definition and measure	Estimate (95% CI)
**RSV LRTI incidence in first six months of life**	Nirsevimab phase 3 trial [25]	Late preterm and term infants, <12 months at baseline (mostly ≤ 3 months), followed up to day 150 (control arm); 20 countries	RSV medically attended LRTI^c^; annualized incidence rate (per 1000)	108 (80–147)
	2022 RSV LRTI systematic review [7]	<6m; global	RSV LRTI; annual incidence rate (per 1000)	96 (68–143)

**Hospitalized RSV LRTI incidence in first six months of life**	Nirsevimab phase 3 trial [25]	Late preterm and term infants, <12 months at baseline (mostly ≤ 3 months), followed up to day 150 (control arm); 20 countries	Hospitalized RSV LRTI; annualized hospitalization rate (per 1000)	32 (18–58)
	2022 RSV LRTI systematic review [7]	<6m; global	RSV LRTI hospitalization; annual hospitalization rate (per 1000)	20 (15–29)

**Severe RSV LRTI incidence in first three months of life**	ResVax phase 3 trial [74]	Newborns followed up to day 90 (control arm); 11 countries (mostly from South Africa and US)	RSV medically significant LRTI^d^; annualized incidence rate (per 1000)	24 (18–34)
	2022 RSV LRTI systematic review [7]	<3m; global	RSV LRTI with chest wall indrawing; annual incidence rate (per 1000)	28 (13–68)

**Hospitalized RSV LRTI incidence in first three months of life**	ResVax phase 3 trial [74]	Newborns followed up to day 90 (control arm); 11 countries (mostly from South Africa and US)	Hospitalized RSV LRTI; annualized hospitalization rate (per 1000)	37 (28–48)
	2022 RSV LRTI systematic review [7]	<3m; global	RSV LRTI hospitalization; annual hospitalization rate (per 1000)	25 (18–37)

**RSV LRTI incidence in first three months of life**	ResVax phase 3 trial [74]	Newborns followed up to day 90 (control arm); 11 countries (mostly from South Africa and US)	RSV LRTI with severe hypoxemia^e^; annualized hospitalization rate (per 1000)	10 (6–16)
	2022 RSV LRTI systematic review [7]	<3m; global	RSV LRTI hospitalization with hypoxemia; annual hospitalization rate (per 1000)	7 (4–16)

**RSV LRTI incidence in first six months of life in low-resource setting**	Motavizumab phase 3 trial [30]	Term infants ≤ 6 months at baseline (mean age: 2 months), followed up to day 150 (control arm); native American	RSV LRTI, inpatient and outpatient; annualized incidence rate (per 1000)	403 (368–441)
	2022 RSV LRTI systematic review [7]	<6m; low- and middle-income countries	RSV LRTI; annual incidence rate (per 1000)	104 (70–154)

**RSV LRTI hospitalization incidence in first six months of life in low-resource setting**	Motavizumab phase 3 trial [30]	Term infants ≤ 6 months at baseline (mean age: 2 months), followed up to day 150 (control arm); native American	RSV LRTI, inpatient only; annualized incidence rate (per 1000)	165 (140–194)
	2022 RSV LRTI systematic review [7]	<6m; low- and middle-income countries	RSV LRTI hospitalization; annual hospitalization rate (per 1000)	19 (13–29)

**RSV LRTI in-hospital case fatality ratios in early childhood in low-resource settings**	PERCH multi-country case-control study [28], [29]	Children aged 1-<60 m; seven countries (mostly low-income)	RSV severe pneumonia in-hospital CFR (%)	2.2 (1.3–3.6)
	2022 RSV LRTI systematic review [7]	<60 m; low-income countries	RSV LRTI in-hospital mortality; CFR (%)	1.4 (0.6–2.8)

**Severe RSV LRTI incidence in first three months of life in low-resource settings**	ANISA observational cohort study [27]	Newborns actively followed to day 59 through active community surveillance; Bangladesh, India, and Pakistan	Possible serious bacterial infection^f^; annualized incidence rate (per 1000)	32 (29–38)
	2022 RSV LRTI systematic review [7]	<3m; lower-middle income countries	RSV LRTI with chest wall indrawing; annual incidence rate (per 1000)	46 (24–86)

**RSV LRTI incidence in early childhood**	IHME GBD 2016 [31]	All ages, <60 months reported as a separate age band; medical records based on clinical databases across the globe	RSV attributable LRTI morbidity; annual episodes in millions	11 (7–17)
	2022 RSV LRTI systematic review [7]	<60 m; global	RSV LRTI; annual episodes in millions	33 (25–45)

**RSV LRTI mortality in early childhood**	IHME GBD 2016 [31]	All ages, <60 months reported as a separate age band; medical records based on clinical databases across the globe	RSV attributable LRTI mortality; annual deaths in thousands	41 (23–66)
	2022 RSV LRTI systematic review [7]	<60 m; global	RSV-attributable deaths; annual deaths in thousands	101 (85–125)

Notes. a) Abbreviations: RSV = respiratory syncytial virus; LRTI = lower respiratory tract infection; CFR = case fatality ratio; PERCH = Pneumonia Etiology Research for Child Health (PERCH) case-control study; ANISA = Aetiology of Neonatal Infections in South Asia (ANISA) observational cohort study; IHME GBD = Institute for Health Metrics and Evaluation (IHME) Global Burden of Disease estimates. b) For each pair of comparison, the best comparable population and case definition from the present study was selected. c) Physical examination findings localizing to lower respiratory tract plus any of the following: 1) fast breathing (≥50 breaths/minute in children aged 2-<6 months); 2) Hypoxemia (SpO^2^ < 95% at ≤ 1800 m elevation); 3) clinical signs of severe respiratory diseases. d) ≥1 LRTI manifestation plus fast breathing (≥60 breaths/minute in children aged > 2 months); or hypoxemia (SpO^2^ < 95% at ≤ 1800 m). e) SpO2 < 92% at ≤1800 m or documented use of supplemental O^2^ or ventilation. f) Based on one of the following signs: fast breathing, hyperthermia, movement only with stimulation, convulsions, and poor feeding; fast breathing cannot be the only sign.

## 4 Preventive interventions

Palivizumab, a humanized monoclonal antibody (mAb) directed against the F protein of RSV, is licensed for use in young children at high risk for RSV disease [12]. The immunoprophylaxis is administered by intramuscular injection monthly throughout the RSV season [12]. The utility of palivizumab is limited by its narrow clinical indication and high price [1], [2], [3]. Safe and effective next-generation RSV preventive interventions that provide increased duration of protection are a critical unmet global health need [1], [2].

**Fig. 1:**
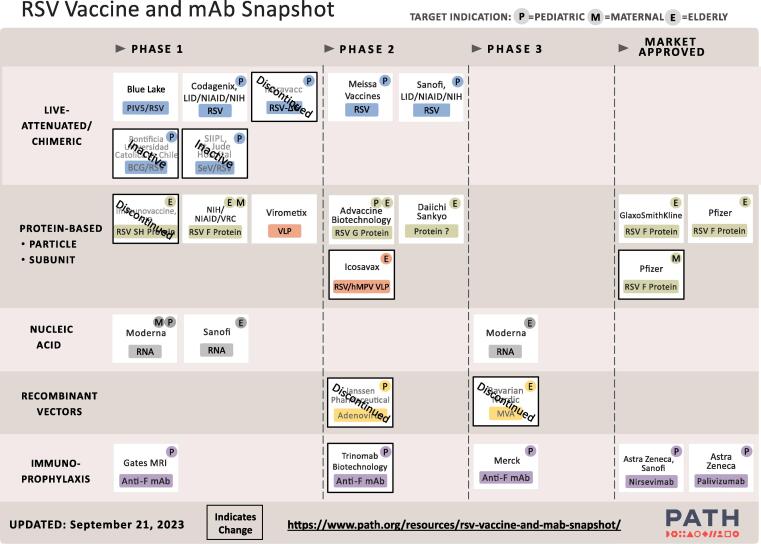
RSV Vaccine and mAb development pipeline. Note: Adapted from the from PATH Clinical Trial Tracker (as of September 21 2023) [13], [32].

At the time of the WHO meeting, there were no licensed next-generation RSV prevention products, although some leading candidates were expected to seek regulatory approval soon. PATH tracks the clinical development landscape of RSV prevention including development stages, target populations, and relevant publications (Fig. 1) [13], [32]. There are three general classes of RSV preventive interventions under development for infant protection: extended half-life mAbs, vaccines for use during pregnancy to protect infants through transplacental antibody transfer, and pediatric vaccines. As of September 2023, extended half-life mAbs and maternal RSV vaccines have been authorized for use in some high-income countries in North America and Europe [14], [33], [34], [35], [36].

Extended half-life mAbs are the first of next-generation RSV prevention products to achieve licensure. Unlike palivizumab, pipeline immunoprophylaxis drugs have an engineered Fc domain with half-life extension crystallizable fragment domain M252Y/S254T/T256E (YTE) mutation, extending circulation to about 70 days, 3-fold that for palivizumab [37]. These drugs could be given as a birth dose or during a later routine childhood immunization timepoint either year-round or before the anticipated RSV season, and they are expected to provide protection through much, or all, of an RSV season [1]. The leading extended half-life mAb candidate, nirsevimab, received market authorization throughout the European Union in November 2022 [14], [38]. In a phase three randomized controlled trial among infants born at gestational age of at least 35 weeks, nirsevimab had an efficacy of 74.5% (95%CI: 49.6%-87.1%) compared to placebo against medically attended RSV LRTI [25]. Similar results were seen in a study of nirsevimab among infants born between 29 and 35 weeks of gestation [26], and nirsevimab protection was comparable to palivizumab among infants with chronic heart or lung disease [39]. Other extended half-life mAbs are under development, including a product by the Bill and Melinda Gates Medical Research Institute with a primary aim for use in LMICs [37].

RSV vaccines for use during pregnancy, like influenza and Tdap vaccines, have been developed for administration during routine prenatal care visits with the primary goal of providing newborns with maternal antibodies against RSV during the first months of life [2]. Maternal vaccines provide protection at the time of birth, unlike pediatric vaccines, and are expected to have lower manufacturing costs than extended half-life mAbs. The exact duration of protection of maternal RSV vaccination is not established, but it is expected to be<6 months, as is seen with maternal influenza and pertussis vaccination [2], [3]. The optimal timing of maternal vaccination is unclear. Current products target vaccination during the late second or third trimester of pregnancy, providing a narrow time window for optimal product delivery [13], [40]. When vaccination does not occur during the third trimester for full term children, or when children are born preterm, product efficacy may be decreased. Further, maternal vaccination platforms will need considerable strengthening before high coverage can be achieved in many LMICs [41]. A Pfizer maternal RSV vaccine candidate has demonstrated higher efficacy (vaccine efficacy against medically attended severe lower respiratory tract illness for 180 days after birth was 69.4%) than modelled by the studies presented here [42]; the results of this trial had not been available at the time of the meeting and the models relied on efficacy results from older trials (see detailed description below). Other vaccine candidates are also in human trials [13]. Pediatric RSV vaccines are in development as well; however, they are not as advanced in clinical development as the other categories [13], and they were not discussed in detail during the meeting.

Despite the limited data on product effectiveness, duration of protection, and prevention coverage, performance goals do exist to inform health economic analyses of RSV prevention. Most notably, WHO has developed Preferred Product Characteristics for RSV maternal vaccines, infant mAbs, and pediatric vaccines [1], [2]. Preferred Product Characteristics describe WHO preferences regarding indications, target groups, immunization strategies, and clinical data for assessment of safety and efficacy. These preferences are shaped by the global unmet public health need in a WHO priority disease area. Other relevant national public health program indicators, such as immunization coverage and antenatal care visit timing and coverage can help estimate RSV product coverage, though they are not wholly interchangeable [43], [44]. The most relevant proxy for birth dose mAb coverage would be coverage for existing birth dose vaccines, including Bacille Calmette-Guérin (BCG) or Hepatitis B virus. Extended Programme on Immunization routine immunization contacts also include visits around 6, 10 and 14 weeks, 9 months, and a timepoint during the second year of life. One of these timepoints could potentially be used for mAb delivery. Seasonal campaign dosing approaches may be programmatically challenging in LMICs where this has not been done for other vaccines. National coverage estimates for routine immunization during pregnancy are limited, so modelers are more likely to use antenatal care coverage estimates as a proxy for maternal RSV vaccination coverage [45].

While the efficacy and duration of protection may not be equivalent across classes of RSV preventive interventions, more product-specific clinical data are anticipated in the next few years to inform estimates of prevention impact in LMICs. Beyond decision making, supporting product delivery—including platforms, logistics, training, and monitoring—will be required for successful introduction, uptake, and ultimately coverage. Finally, product acceptability is a critical input and may differ between interventions, location, and across time.

## 5 Cost-effectiveness studies in LMICS

At the WHO-sponsored meeting, four cost-effectiveness studies for RSV prevention in LMICs were reviewed—one each considering cost-effectiveness for 72 Gavi-eligible countries [46], 131 LMICs [47], and Mali [48], and a joint analysis for Kenya and South Africa [49] (Table 2). These studies all used static models to estimate RSV LRTI health outcomes and costs. The ages of children varied from the first six months to the first five years of life. Each measured health impact in disability adjusted life-years (DALYs) and costs in US dollars with a discount rate of 3% applied to future health and economic outcomes. DALYs are a widely-used metric that combine years of life lost from mortality with years of healthy life lost from morbidity and they are a standard way to express health impact in cost-effectiveness studies as they can be compared across disease states and etiologies.

**Table 2:** Parameter inputs from RSV prevention cost-effectiveness analyses in low- and middle-income countries.

	Li et al 2020 [46]	Laufer et al 2021 [48]	Baral et al 2020 [75]	Koltai et al 2022 [49]
**Location**	72 Gavi-eligible countries	Mali	131 LMICs	Kenya and South Africa

**Model type**	static	static	static	static

**Age Inclusion (years)**	0–5	0–0.5	1	0–5

**Time horizon (years)**	5	0.5	10	5

**RSV incidence rate**	NA	age- and month-specific (mean = 53.7%)	NA	Age- and country-specific (monthly resolution under 1 year) of ARI and SARI, medically attended or not

**RSV LRTI incidence rate**	Age- and country-specific (monthly resolution under 1 year; country rates from 3.5 to 6.7%)	NA	age-specific (4% − 9.96%)	Age- and country-specific (monthly resolution under 1 year)

**RSV hospitalization incidence rate**	NA	NA	NA	Age- and country-specific rates of hospitalized and non-hospitalized SARIs

**Probability of LRTI given RSV**	NA	0.13	NA	NA

**Probability of inpatient care given RSV LRTI**	0.09	0.29	20.2 per 1000 for 0–5 months, 11 per 1000 for 6–11 months	Age-specific hospitalization rates (<1 year: 5–60 hospitalizations/1000 population)

**Hospital case fatality rate**	age-specific (0.045–0.006)	0.016	0.022 for 0–5 months, 0.024 for 6–11 months	Age-specific mortality rates (under 1 year: 25–150 deaths/100.000 population)

**Disability weight, severe RSV LRTI**	0.21	0.13	0.21	0.21

**Disability weight, moderate RSV LRTI**	0.053	0.05	0.053	0.053

**QALY loss, severe RSV LRTI**	NA	NA	NA	NA

**QALY loss, moderate RSV LRTI**	NA	NA	NA	NA

**Duration of illness (days)**	11.2	8.5	10 for severe RSV LRTI, 5 for moderate RSV LRTI	11.2

**Life expectancy (years)**	country-specific (50–80)	58	country-specific (50–80)	Kenya: 66.5, South Africa: 62.5

**Discount rate (%)**	3	3	3	3

**Currency**	2016 USD	2019 USD	2016 USD	2021 USD

**Willingness to pay threshold (USD per DALY averted)**	continuous (0–30000)	891	country-specific (130–4774)	not fixed

**WTP as a multiplier of country GDP per capita**	NA	1	0.5	NA

**Outpatient costs (USD)**	country-specific (0.13–91)	6.56	53	Kenya: 20.9 USD, RSA: 24.95 USD

**Inpatient costs (USD)**	country-specific (0.37–640)	118.57	250	Kenya: 102 USD for healthcare provider +172 USD for household (out-of-pocket); RSA: 634–1002 USD for healthcare provider +4–22 USD for household (out-of-pocket)

**ICU costs (USD)**	NA	NA	NA	NA

**Administration cost per dose (USD)**	included in intervention cost per dose	1.35	0.63 for LIC, 1.73 LMIC and UMIC	included in intervention cost per dose

**Cost per dose, short-acting mAb (USD)**	NA	3	NA	NA

**Cost per dose, long-acting mAb (USD)**	6 (tested value: 4 and 11)	3	3 for Gavi eligible, 5 for non-Gavi	Tested values: 6, 20, 60

**Cost per dose, maternal vaccine (USD)**	3	3	3 for Gavi eligible, 5 for non-Gavi	Tested values: 3, 10, 30

**Outcome efficacy protects against**	RSV LRTI cases	RSV cases	RSV LRTI cases	RSV LRTI, RSV LRTI with hospitalization, severe RSV LRTI (death)

**Efficacy, short-acting mAb (%)**	NA	78	NA	NA

**Efficacy, long-acting mAb (%)**	70 (tested value 50 and 90)	56	60–70	70.1%, 78.4%, 78.4% [no data for efficacy against deaths]

**Efficacy, maternal vaccine (%)**	70 (tested value 50 and 90)	70	40–60	39.4%, 44.4%, 48.3% (the efficacy figures were updated in the published version of the article, lowering the ICER values [49])

**Efficacy, pediatric vaccine (%)**	NA	NA	NA	NA

**Duration of protection, short-acting mAb (months)**	NA	1	NA	NA

**Duration of protection, long-acting mAb (months)**	6 (tested value: 4 and 8)	5	6	5

**Duration of protection, maternal vaccine^a^ (months)**	5 6 (tested 6alue: 3 and 8)	3	3	3

**Coverage^b^, short-acting mAb (%)**	NA	77	NA	NA

**Coverage^b^, long-acting mAb (%)**	country-specific (52–99)	83	82	95%

**Coverage^b^, maternal vaccine (%)**	country-specific (52–99)	35.5	84	95%

**ICER^c^, short-acting mAb**	NA	4280	NA	NA

**ICER^c^, long-acting mAb**	country-specific (3152–7927)	1656	431	6 USD dose price: Kenya: 325 South Africa: cost-saving 60 USD dose price: Kenya: 6248 South Africa: 5583 USD

**ICER^c^, maternal vaccine**	country-specific (1708–5663)	8020	1342	3 USD dose price: Kenya: 734 South Africa: cost-saving 30 USD dose price: Kenya: 10,186 South Africa: 10,099

Notes: a) Duration of protection for maternal vaccine begins at birth. b) Coverage refers to percentage receiving intervention among those eligible. c) Units for ICERs are USD per DALY averted.

While each study examined the expected health and economic impact of extended half-life mAb and RSV maternal vaccine, they used different assumptions regarding intervention efficacy, duration of protection, and product cost. In general, extended half-life mAbs are estimated to have lower incremental cost-effectiveness ratios (indicating higher value for money) than equally priced RSV maternal vaccine. As the price of mAb rises relative to maternal vaccine, maternal vaccine becomes increasingly more favorable. Seasonal administration of mAb limited to the months of highest RSV risk also improves the value for money compared to year-round administration. A seasonal strategy is advised by the PPC in settings where the RSV season is clearly defined [1]. Only the Mali study considered a seasonal program, which contributed to the more favorable cost-effectiveness ratio for mAb in that analysis.

**Fig. 2:**
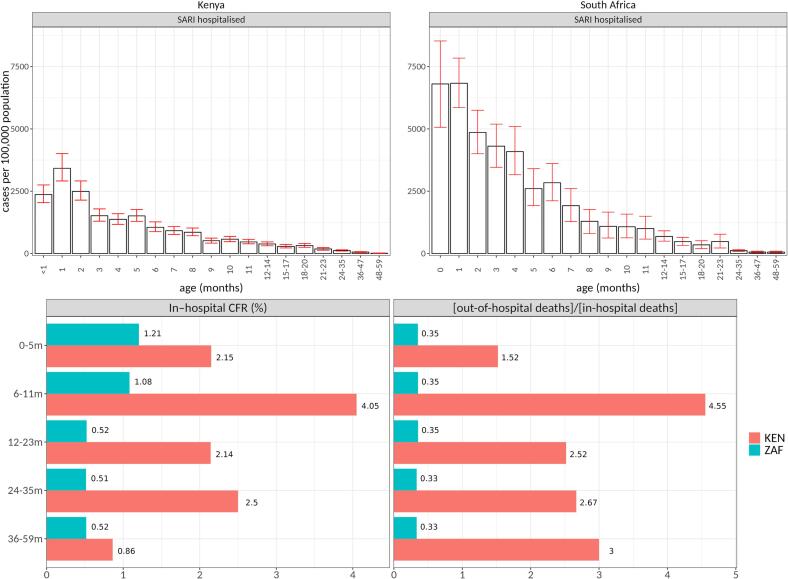
Hospitalized SARI cases, in-hospital CFR values and the estimated ratio of out-of-hospital to in-hospital deaths in Kenya and South Africa. Note: As the overwhelming majority of the RSV disease burden in children under the age of 1 in Kenya and South Africa is estimated to be due to RSV-associated deaths, the parameters that most strongly influence the burden reduction are the age-specific CFR of in-hospital and out-of-hospital severe cases and the efficacy and duration of RSV preventive interventions against severe RSV LRTI. More deaths within the window of effectiveness of the RSV preventive interventions will lead to a proportionally larger reduction in the total disease burden. A longer duration or higher efficacy of the effect against deaths will similarly lead to a proportionally larger reduction of the burden and thereby lower the DALYs averted, improving the cost-effectiveness of the interventions. The dose price of RSV preventive interventions will scale the cost-effectiveness of the interventions linearly. Figure reproduced from a previous publication [49].

Data from Kenya and South Africa reveal that RSV LRTI incidence and death are concentrated among infants in the first three months of life [49], whereas in Mali RSV LRTI incidence was greatest in the fourth and fifth months of life [48]. For this reason, cost-effectiveness estimates for maternal vaccine aimed at protection during early infancy were more favorable in Kenya and South Africa compared to Mali. Whether these differences in age distribution of early RSV disease are due to true differences in epidemiology, health care utilization or in surveillance approaches is not clear. However, the impact of this discrepancy on intervention cost-effectiveness highlights the importance of robust estimates of early-life RSV epidemiology and health-care utilization within regions and countries. Additionally, as deaths are the largest driver of DALYs averted, RSV case fatality rates in the hospital and in the community are critically important inputs. Both large multi-country studies applied an adjustment factor of 2.2 to all country-specific inpatient case fatality rates to estimate the rate of community deaths [23], [46], [47]. In the Kenya and Mali analyses, deaths in the community accounted for approximately 3/4 of all RSV-associated deaths, whereas in South Africa they made up about a quarter (Fig. 2) [49]. It is possible that these studies have underestimated the total number of RSV associated deaths, as the 2022 systematic review of RSV LRTI burden estimates suggests approximately four community deaths for each in-hospital death in low-income countries [7].

**Fig. 3:**
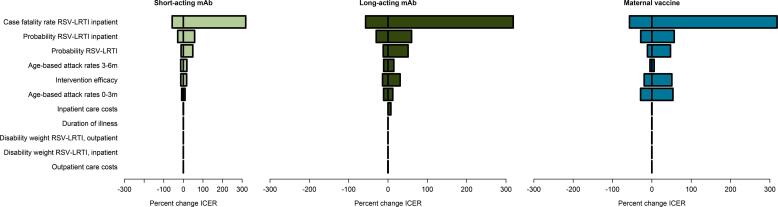
A) Univariate sensitivity analysis for Mali. Note: A series of univariate sensitivity analyses were conducted to assess the parameters whose variance has the largest influence on cost-effectiveness estimates for Mali. The parameter with the largest influence on the ICER across interventions is the inpatient case fatality rate (>300%). Parameters with moderate (<60%) influence include the probability of being hospitalized with RSV LRTI, probability of LRTI given RSV, age-based RSV attack rates, intervention product efficacy, and inpatient care costs. As deaths have the largest impact on cost-effectiveness estimates, case fatality rates are critically important inputs to capture accurately. Figure reproduced from a previous publication [48].

**Fig. 4:**
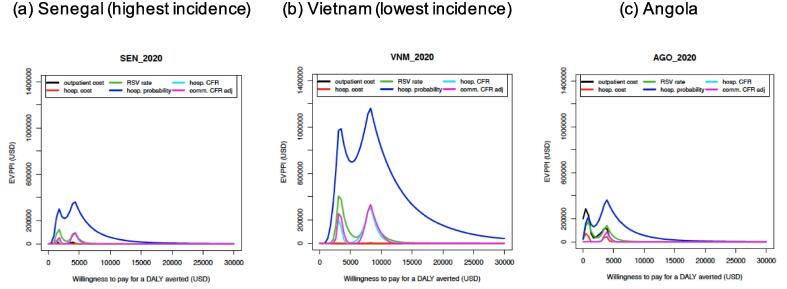
Expected Value of Partially Perfect Information for Senegal (high incidence), Vietnam (low incidence), and Angola. Note: In Fig, three examples are presented to demonstrate the influential factors. The age-specific RSV hospitalization probability is the most influential factor for all countries. RSV incidence rate, hospital case-fatality ratio and community case-fatality ratio are also top influential factors. A few countries (like Angola) show that cost of outpatient care is an influential factor at low willingness-to-pay level (<1000 USD per DALY averted), because the cost of outpatient care is higher and more uncertain compared to other countries. However, at higher WTP levels, the top-ranking influential factors are the same as the other countries. Figure reproduced from a previous publication [46].

Assessing model sensitivity to either different assumptions or changing conditions is critical to understanding the decision space, or in other words, which model changes might lead to a different policy choice. Univariate sensitivity analyses, in which individual parameters are varied incrementally above and below a point estimate, can identify which parameters most influence model output (Fig. 3). Another important analysis tool for decision models is the Expected Value of Partially Perfect Information, which calculates the amount that key stakeholders would be willing to spend to gain an exact estimate for a specific influential parameter. The Expected Value of Partially Perfect Information is calculated as the difference in the monetary value of health gain associated with a decision made using the currently available information and when the choice is made based on perfect information without uncertainty (Fig. 4) [50]. Among the studies presented at the meeting which assessed parameter influence, the authors identified rates of illness, hospitalization, and death due to RSV as the most influential. Identifying influential parameters can help to determine target areas for funding further research and data collection, especially when expensive trials and observational studies are involved.

## 6 Key parameters for RSV prevention cost-effectiveness

### 6.1 Cost of care

Few primary data collection studies have been done on the cost of facility treatment specifically for RSV, with general pneumonia costs often used as proxies [51]. Additionally, there is a paucity of data regarding intensive care unit (ICU) and ventilation costs among RSV patients. However, facility treatment costs for RSV may not be the most influential drivers of the cost-effectiveness of RSV interventions in low-resource settings, due to the often-low cost of care and healthcare utilization [46]. Most of the economic benefits from RSV interventions derive from the value of prevented mortality (DALYs averted), which may be relatively higher in such settings partly because of low healthcare access. Rates of facility treatment may grow over time if countries are able to invest more in healthcare systems as a whole. Under these conditions, the costs averted by preventive RSV interventions will increase; this may even make RSV interventions net cost saving as suggested by the cost-effectiveness results for South Africa [49].

RSV preventive interventions may also achieve broader cost savings apart from direct healthcare expenditures, which are less commonly measured. Costs for out-of-pocket payments, transport, accommodation, and lost productivity may fall on households of infants with RSV illness; these were measured in a study of RSV hospitalization in Malawi [52]. Studies in high-income countries suggest that the productivity costs can last well beyond the acute episode itself [53]. RSV illness has been associated with long-term sequelae such as wheezing and asthma [54]; if these can be prevented by vaccination or mAbs then the long-term medical and productivity cost savings may be substantial. Antibiotics are often inappropriately prescribed to treat respiratory illness associated with RSV [55]. Hence RSV preventive interventions may reduce both the costs of antibiotic prescribing and the long-term costs and health losses associated with the loss of antibiotic efficacy due to overuse. The studies discussed at the meeting did not include these cost elements, and therefore are likely underestimating the full societal value of RSV interventions.

### 6.2 Age specific CFR of RSV LRTI

Because mortality is a primary driver of the cost-effectiveness ratio for RSV preventative interventions in LMICs, it is critical that it be estimated as accurately as possible. Despite progress in updating global RSV mortality estimates using rigorous methodology [7], the number of studies directly measuring RSV deaths in LMICs remain few and are faced with several inherent challenges. Three such challenges include (1) estimating the proportion of deaths with RSV detected that are caused by RSV, i.e., differentiating RSV-attributable from RSV-associated deaths; (2) estimating the number of deaths in LMICs that occur outside of health facilities; and (3) estimating the out-of-facility RSV CFR, which likely is higher than the in-hospital CFR.

The presence of RSV in a deceased child, identified through antemortem or post-mortem sampling (i.e., an RSV-associated death), does not always indicate that the death was attributable to the RSV infection. Using RSV-associated deaths to estimate CFR can therefore lead to over-estimates of the mortality that could be prevented by RSV-targeted interventions, and therefore an inaccurate cost-effectiveness assessment. Conversely, RSV could be in the causal chain leading to death and no longer be detectable once samples are obtained, leading to under-estimation of its role. Differentiating RSV-associated from RSV-attributable illness and death can be complicated, as multiple pathogens are often detected from the same LRTI episode [56]. Although there is compelling evidence that RSV is causally associated with LRTI episodes when it is detected in a child with LRTI, it is not clear that detecting RSV in fatal cases is similarly predictive of death caused by RSV [56], [57]. This is highlighted by the Child Health and Mortality Prevention Surveillance Study (CHAMPS), a multi-site study where expert panels determine cause of death from post-mortem specimens, verbal autopsy and antemortem clinical records. In pooled cases from CHAMPS sites representing seven countries, RSV was determined to be in the causal chain leading to death in 24 cases among 67 where it was detected (36%), with considerable variation by age group and study site [58]. The implication is that mortality could have been prevented by RSV-targeted intervention in only 1/3 of these RSV-associated deaths.

A second major challenge is estimating the proportion of RSV deaths in children that occur outside of health care facilities. This is a particularly important consideration for low resource setting with a high burden of deaths from all causes, including RSV, in the community. Community mortality studies in infants < 6 months document a high proportion of RSV deaths occurring in the community, ranging from 29% in Karachi, Pakistan to 70% in Lusaka, Zambia to 75% in rural Maharashtra, India [59], [60], [61]. These figures may be over-estimates based upon the possibility that RSV might not have been in the cause chain of death in some of the decedents where it was identified.

A third challenge is estimating the CFR for RSV illness that occurs in the community. In Maharashtra, community and in-hospital CFRs were directly compared for the same cohort of children < 6 months [60]. In this cohort, community RSV CFR was 2.5 times greater than the in-hospital RSV CFR (3/52 [7.1%] vs. 1/36 [2.8%]). Although limited by small numbers, this study demonstrates that applying in-hospital RSV CFR to community incidence may underestimate community mortality.

The methodologic, logistic, and ethical barriers to generating accurate RSV-attributable mortality estimates and CFRs in low resource settings are significant. These inputs will therefore be most reliably generated with post-introduction studies of RSV vaccines or mAbs [62].

### 6.3 RSV intervention product pricing and delivery costs

Immunization program costs are comprised of commodity costs and delivery (i.e., administration) costs. To date, there are limited data to directly inform the costs of RSV intervention programs, as only limited interventions are available. Commodity prices are not yet known, and delivery costs are only now beginning to be assessed. However, some information can be inferred from other vaccines and associated delivery costs. Broadly speaking, RSV vaccine commodity costs are likely to depend on the complexity of developing and manufacturing the product, market size and makeup (i.e., potential for different market segments), number and location of suppliers, country income level or ability to pay, donor support, and time since the intervention has entered the market. These commodity costs are thus linked to supplier-related costs and other market factors that will also influence prices. Delivery costs are likely to be influenced by country income level, delivery strategy and ability to leverage other program activities. These factors can help interpret data from other vaccines that might serve as proxies as RSV specific information is forthcoming.

Product pricing for currently available vaccines can be assessed through several sources including the UNICEF and WHO websites [63], [64]. Data from UNICEF show that product prices can vary substantially by vaccine and may even differ substantially even within a single product. For example, average prices for measles vaccine, oral polio vaccine (OPV), or diphtheria-pertussis-tetanus vaccine may cost less than $0.25 per dose. Other newer products or those with markets dominated by multinational producers such as human papillomavirus vaccine or pneumococcal conjugate vaccine may command higher prices. Prices can also vary depending on the procurement mechanism and country income level. Between 2018 and 2020, average country reported prices for Prevnar13 varied substantially. Countries eligible for Gavi support reported prices approximating $3.50 per dose while countries procuring through the Pan American Health Organization (PAHO) revolving fund paid approximately four times this amount. Average reported prices were slightly higher than PAHO revolving fund prices for other lower- and upper-middle income countries [64]. On average, high-income countries reporting prices paid nine times the average price paid by countries eligible for Gavi support. Country income level and donor support are important factors influencing vaccine prices. While prices for RSV prevention interventions are not yet known, similar trends may be expected when these products come to market.

To date, there are no known studies assessing RSV intervention delivery costs, though several prospective studies are being initiated. As with product price, information can be gleaned from other vaccines to inform potential delivery costs. The Immunization Delivery Cost Catalogue and associated publications are a useful source of delivery cost data [65]. While delivery strategy, study method, country context and other factors limit direct comparability, most studies find that the economic cost to deliver a vaccine ranges from approximately $0.50 to $1.50 USD. However, costs for human papillomavirus vaccine delivery can be higher due to the potential for alternative delivery strategies to reach a different target population through unique contacts with recipients. Maternal immunization may also require alternative delivery strategies, unique contacts with recipients or seasonal delivery and thus may cost more to deliver. There are currently few empirical estimates of maternal immunization delivery costs in LMICs, though existing estimates broadly align with estimates for childhood vaccines [66].

Prospective RSV or maternal immunization delivery cost estimates will help inform our understanding of whether maternal immunization delivery costs will align with existing childhood vaccine delivery costs or if they may cost more due to distinct contacts with beneficiaries, alternative delivery strategies or platforms, e.g., integration with antenatal care programs. There are no known estimates of mAb delivery costs in LMICs, but these costs may be similar to other childhood vaccines. Our knowledge of RSV intervention program costs is limited but expected to grow quickly as RSV preventive interventions become available and enter use.

### 6.4 Willingness to pay for health

Once a cost-effectiveness ratio has been estimated, the result must then be interpreted for policy decisions. The amount of money that an entity will spend in order to achieve a unit of improved health for a given population under its remit is often referred to as the societal willingness to pay, or as the cost-effectiveness threshold [67]. The WHO Choosing Interventions that are Cost-Effective (CHOICE) Programme offers guidance for evaluating new interventions, centered on comparison with existing interventions and alternative spending choices. Under this framework, the maximum willingness-to-pay for health might be approximated as the highest cost-effectiveness ratio for a currently funded intervention that is deemed cost-effective, with the caveat that cost-effectiveness is not the sole consideration when selecting health programs [68]. Previous documents suggested designating “very cost-effective” and “cost-effective” interventions for a country based on per-capita gross domestic product (GDP) and three times that value, respectively [69]. These numbers were widely adopted as global norms in cost-effectiveness analyses [68], and have often been used as a decision rule, despite replacement with new guidance as well as evidence that these thresholds may be unrealistically high for LMICs [70].

The willingness to pay intersects with cost-effectiveness and policy decisions in ways that are both intuitive and not. Intuitively, as the willingness to pay rises, higher cost-effectiveness ratios become acceptable to payers. Interventions become more likely to be adopted, and higher prices better tolerated. When there are multiple payers, this general principle remains true, but each payer may end up preferring different decisions or strategies. For instance, a donor generally will have a higher willingness or ability to pay for health than a recipient, by nature of their relationship. A donor who is subsidizing an intervention across multiple countries may also be less sensitive to the cost-effectiveness of the program in a single country, and willing to accept high cost-effectiveness ratios for some contexts when the overall value for health is favorable. Another aspect of the donor/recipient dynamic is that cost-sharing may lead to different cost-effectiveness ratios for each payer and potentially different policy preferences. For instance, under a donor model similar to that used by Gavi, combination strategies using both extended half-life mAb and pediatric vaccination have a lower cost-effectiveness ratio from a government payer perspective than a donor perspective in Mali [71]. However, if the donor willingness-to-pay is higher than that of the government, this combination strategy might be optimal from both perspectives [72].

### 6.5 Summary of the discussion about key parameters

Objectives of the meeting included identifying the most influential parameter inputs and data limitations for the cost-effectiveness analyses and recommending and prioritizing future data gathering and research to improve estimates of the impact of RSV prevention in LMICs. Epidemiological parameters from the presented health economics studies identified as both influential and uncertain were those associated with RSV hospitalization and death, specifically setting-specific hospitalization rates and RSV-attributable death rates. Influential economic parameters included product price, delivery costs, willingness-to-pay for health on the part of potential donors, and the cost of RSV-associated hospitalization. Participants appraised the research presented in the meeting as being of high quality, with the caveats that the health economics studies used inputs for which there were limited empiric data. Public health donors and investigators should consider future research to develop more robust, precise measurements of the parameters identified by the meeting as influential and uncertain.

The most influential disease epidemiology data include incidence of severe and fatal RSV LRTI. These relatively rare endpoints are difficult to measure precisely with most observational study designs. Pooling data from multiple studies for *meta*-analysis is the most efficient way to address the issue of lack of power, and standardized case definitions and data collection procedures could facilitate these efforts. Further, vaccine or mAb probe design may be able to reveal the fraction of hospitalizations that are attributable to RSV and thus preventable through product use.

It is anticipated that more product-specific data, such as duration of protection and efficacy from LMIC settings will become available as field trials progress. Additional valuable data can be achieved from observational effectiveness studies. Standardization of case definitions, methodologies, data reporting can facilitate study-to-study comparisons and data pooling.

This meeting highlighted the limitations in the availability of general LRTI or RSV-specific medical care costs, as well as costs related to product delivery. More data collection from diverse locales would benefit impact models.

## 7 Discussion

As RSV preventive interventions move through clinical development towards licensure, there is an urgent need to consider the suitability of these products for use in LMICs. Palivizumab is unsuitable due to its price point and the need for multiple doses. Products meeting WHO Preferred Product Characteristics would have lower barriers: a single-dose maternal vaccine, a two-dose pediatric series, or a birth dose mAb with extended half-life. For high-income countries where the short half-life monoclonal is currently used, the health economic case for next generation products may be straightforward. At a similar or lower price and with higher protection, these products can replace the short half-life mAb and could be offered to all infants. However, in LMICs the adoption of these strategies represents a substantial financial outlay that may not be entirely offset by savings on medical care. The cost-effectiveness of these new strategies will be a critical consideration for public health policymakers aiming to maximize health with limited resources.

In convening this meeting, we aimed to illuminate the known drivers of cost-effectiveness for these interventions based on existing health economic models, and to highlight where insufficient knowledge contributes to uncertainty regarding the appropriate public health decision. We also sought to clarify the factors contributing to cross-country variability in parameter estimates. Finally, it was our goal to identify whether there was a clear need for future research to resolve these uncertainties.

The first major challenge is accurate determination of the health burden that could be alleviated by each prevention strategy. In most LMICs, RSV illness data remains scarce. Disease burden estimations often rely on sentinel sites or research studies to extrapolate information across broad geographic areas and populations. Complicating quantification, recent studies suggest that some proportion of deaths among RSV-positive infants which occur in a hospital setting are likely attributable to a different pathogen or cause, and therefore could not have been prevented by any of these RSV-specific preventative products [29]. As a further complication, evidence indicates that more RSV deaths than previously suspected occur in the community [7] and are not documented at a hospital setting. These biases pull the estimates of disease burden in opposing directions, adding considerable uncertainty.

The investment case for RSV preventive interventions also relies on economic inputs such as the costs for medically attended RSV illness. There may not be substantial uncertainty at the country level; for instance, assessment of RSV prevention in Mali using high-quality, setting-specific inputs found that even relatively wide ranges for medical costs did not lead to large changes in the economic case for RSV prevention [48]. However, variation across countries can dramatically change the decision space. In South Africa, for instance, greater healthcare utilization and higher costs for RSV illness leads to the conclusion that RSV prevention strategies could be cost saving for that country [49]. International decision-making bodies and donors must be aware of these cross-country drivers, so that a less favorable cost-effectiveness ratio is not necessarily interpreted as due to a lower disease burden, but potentially to greater investment in, and access to, healthcare.

Changes across reasonable ranges for the product price and willingness-to-pay for health also influence whether these RSV prevention strategies would be considered favorable or unfavorable. As the vaccine-preventable mortality is lower for RSV than for other pathogens such as *Haemophilus influenzae* type B [73], acceptable prices for RSV preventive interventions are also lower than for these vaccines. It is not yet clear whether these lower prices are feasible for manufacturers, particularly for mAbs. Regarding the willingness-to-pay for health, WHO and other global bodies have moved away from single yardsticks for cost-effectiveness. The previous commonly used measures of one and three times the per-capita GDP per DALY averted may not reflect true budget constraints, which may cap the interventions that could efficiently be adopted at a lower range. For example, in the analysis of RSV prevention in Mali, the authors found that extended half-life monoclonals have an incremental cost-effectiveness ratio (ICER) of approximately US $200 per DALY from the government perspective, which would generally be considered good value even with this new perspective [48]. However, the societal and donor ICERs are twice and three times higher, respectively. Although it is reasonable to expect that donors might be willing to pay for interventions that are not otherwise affordable, as that is the nature of donation, it is not clear whether donors value health at ICERs in these specific ranges.

## 8 Conclusion

RSV LRTI is a major cause of death and suffering among young children in LMICs. Prevention of RSV LRTI is a major unmet need in these settings. There is a robust pipeline of RSV preventive intervention candidates in clinical development, including an extended half-life mAb recently authorized for use in Europe and a maternal vaccine undergoing regulatory review. Vaccine decision makers will need estimates of cost effectiveness to inform policies and implementation. These cost-effectiveness estimates will require data that are not routinely collected through public health practice nor in intervention efficacy studies. This meeting identified the most influential modelling parameters which could drive results about intervention cost effectiveness. Precise and high-quality estimates for these parameters will improve health and economic impact estimates of RSV prevention.

**Disclaimer:** The authors alone are responsible for the views expressed in this article and they do not necessarily represent the views, decisions, or policies of the institutions with which they are affiliated.

**Funding:** This meeting was funded by a grant from the Bill & Melinda Gates Foundation (Global Health Grant OPP1114766) to the World Health Organization, which sponsored the meeting.

## Declaration of Competing Interest

The authors declare the following financial interests/personal relationships which may be considered as potential competing interests: [Meagan C. Fitzpatrick: received grants to her institution from the National Institutes of Health, National Science Foundation, World Health Organization, and Bill & Melinda Gates Foundation; consulting fees from Sanofi Pasteur and The Commonwealth Fund. Rachel S. Laufer: none to declare. Ranju Baral: none to declare. Amanda Driscoll: none to declare. Danny Feikin : none to declare. Jessica A. Fleming: none to declare. Mark Jit: Mark Jit is an unpaid member of the Respiratory Syncytial Virus Consortium in Europe (RESCEU) and Preparing for RSV Immunisation and Surveillance in Europe (PROMISE). RESCEU and PROMISE have received funding from the Innovative Medicines Initiative 2 Joint Undertaking. This Joint Undertaking receives support from the European Union’s Horizon 2020 research and innovation program and the European Federation of Pharmaceutical Industries and Associations. Neither MJ nor his research group has received any forms of pecuniary or other support from the pharmaceutical industry. Sonnie Kim: none to declare. Mihaly Koltai: none to declare. You Li: Grants to his institutions from Wellcome Trust and GSK; personal fees from Pfizer, all outside the submitted work. Xiao Li: none to declare. Harish Nair: Received funding from Innovative Medicines Initiative, National Institute of Health Research, Pfizer, and Icosavax; Consultancies from Sanofi, Pfizer, GSK, MSD, ReViral, Icosavax, Astra Zeneca, and Abbvie all outside submitted work. Kathleen M. Neuzil: Is a member of the WHO Strategic Advisory Group of Experts on Immunization. Clint Pecenka: none to declare. Erin Sparrow: none to declare. Padmini Srikantiah: none to declare. Justin R. Ortiz: Grants to his institution from the National Science Foundation, Bill & Melinda Gates Foundation, Pfizer, NIH, and World Health Organization; consulting fees from Putnam and GSK; and participation on advisory boards for Pfizer, Seqirus, and Moderna, all outside the submitted work].

## Data Availability

No data was used for the research described in the article.
